# Editorial: Antithrombotic Prophylaxis in Patients With Atrial Fibrillation for Stroke Prevention: Evidence From Randomized Clinical Trials, Observational Studies, Methodological Modeling, and More

**DOI:** 10.3389/fmed.2021.658303

**Published:** 2021-02-15

**Authors:** Junguo Zhang, Lehana Thabane, Ziyi Li, Guowei Li

**Affiliations:** ^1^Center for Clinical Epidemiology and Methodology, Guangdong Second Provincial General Hospital, Guangzhou, China; ^2^Department of Health Research Methods, Evidence, and Impact (HEI), McMaster University, Hamilton, ON, Canada

**Keywords:** antithrombotic prophylaxis, stroke prevention, atrial fibrillation, direct oral anticoagulants, treatment

Atrial fibrillation (AF) as the most common sustained cardiac arrhythmia, is associated with a 5-fold increase in stroke risk. Use of anticoagulants remains the mainstay to prevent AF-related strokes (1). Since 2009, the direct oral anticoagulants (DOACs), including direct thrombin inhibitor (dabigatran) and factor Xa inhibitors (apixaban, rivaroxaban, and edoxaban), were developed to replace vitamin K antagonists (VKA) for patients with AF, mainly owing to their predictable and favorable pharmacological profiles (2). Nevertheless, with evolving knowledge landscape, obtaining or determining optimal information or best available evidence to guide clinical decision-making for the use of anticoagulants remains a challenge. This special issue has been dedicated to disseminating new insights on the antithrombotic prophylaxis in patients with AF for stroke prevention. Articles outlined below illustrate the most up-to-date evidence of antithrombotic prophylaxis and stroke prevention, which involves several topics to help with clinical practice and patient management including stroke and bleeding risk assessments, the use of DOACs in older individuals, indirect comparison among DOACs in patients with venous thromboembolism (VTE), minimally interrupted DOAC for AF ablation and antithrombotic treatment strategies in patients with AF undergoing elective percutaneous coronary intervention (PCI).

## Stroke and Bleeding Risk Assessment

In their narrative review, Ding et al. discussed concepts and controversies from current evidence of risk factors for stroke and bleeding assessment in AF. They comprehensively summarized a variety of clinical, electrical, biological, and genetic markers used to guide assessments in AF. Nevertheless, the interplay of the involved risk factors was challenging and less well-studied. Currently, CHA2DS2-VASc score remains to be widely recommended to aid in decisions of anticoagulation therapy. Moreover, stroke and bleeding risk stratification should be undertaken by clinicians as a continuous process with specific focus on preventing the development of additional risk factors.

The mini-review by Wu et al. compared the anticoagulant effect of warfarin with DOACs and emphasized the advantageous benefit-harm profiles of DOACs in AF. However, given the lack of long-term monitoring and unavailability in some regions or countries currently, assessing stroke and bleeding risks for decision-making and applying results from large-scale randomized controlled trials to real-world clinical practice became largely difficult in replacing warfarin with DOACs in patients with AF.

## DOACs for Older Patients with AF

Advanced age as one of the well-known risk factors for AF, can also substantially increase risks of stroke and/or systemic embolism (3). Even given a high risk of major bleeding in antithrombotic prophylaxis in the elderly age ≥75 years, anticoagulants own a positive net benefit due to a significantly reduced risk of stroke (4). However, there is a lack of recommendations and guidelines for the use of DOACs in elderly patients. Deng et al. systematically searched three databases and included five randomized controlled trials involving 28,137 elderly participants. They conducted a network meta-analysis to evaluate the efficacy and safety of DOACs vs. warfarin in the elderly. It was found that DOACs had significantly lower risks of stroke and/or systemic embolism and major bleeding, among which apixaban seemed to show a best benefit-harm profile in AF. These findings provide decision aid to support the use of DOACs in elderly patients with AF.

## DOACs for Patients with VTE

VTE is the third most common cause of vascular death after stroke and myocardial infarction. Recent guidelines recommended DOACs for the treatment of VTE; however, they did not suggest a specific DOAC with a most favorable benefit-harm profile. Based on an indirect comparison method, Li et al. found no statistically significant differences among DOACs in terms of efficacy and safety in patients with VTE. Albeit not statistically significant, apixaban tended to have the lowest risk of major bleeding while rivaroxaban had a smallest risk of VTE. It should be noted that the results based on indirect comparisons necessitated extremely cautious interpretation because of potential and unquantified bias, requiring more high-quality evidence to decide which DOAC as the optimal choice for VTE.

## Minimally Interrupted DOAC for AF Ablation

While catheter ablation is a well-established treatment for paroxysmal AF, optimal periprocedural anticoagulation protocols to minimize risk of complications are still largely debated. Tang et al. conducted a retrospective single-center study to evaluate the safety and effectiveness of minimally interrupted DOACs for AF ablation. They found that minimally interrupted DOACs during periprocedural period appeared safer and equally effective when compared with the bridging heparin and uninterrupted VKA therapy, after analyzing 4,520 patients receiving AF ablation. Results from their study offer new insights into determining evidence-based anticoagulation strategy in AF ablation in real-world clinical practice.

## Antithrombotic Therapy in Patients with AF Undergoing PCI

Patients with AF undergoing PCI would generally receive triple antithrombotic therapy (TAT), as recommended by current guidelines, which raises concerns about increased risk of major bleeding. Therefore, dual antithrombotic therapy (DAT) has been suggested to be a preferred option in patients with indication for oral anticoagulation (5). Heger et al. conducted a single-center retrospective cohort study to explore trends of antithrombotic therapies in patients with AF undergoing PCI. They found that duration and prescription of TAT and VKA decreased when compared to DAT with NOAC, and patients receiving treatments with WKA had higher bleeding risk and more co-morbidities. These findings reflect the current real-world practice. However, further large studies from external sources are needed to validate the results and explore the related factors.

In summary, this collection not only highlights the advantages of DOACs in different types of patients, but also provides new insight into optimal intervention selection for risk assessment, therapeutic intervention and disease management in patients with AF. The results from studies in this collection aim to expand our knowledge base on the current clinical practice and the evidence-based options for clinical decision-making for antithrombotic prophylaxis in AF.

## Author Contributions

JZ and GL: conception and design. JZ, LT, ZL, and GL: drafting the article. JZ, LT, ZL, and GL: revising it for intellectual content. JZ, LT, ZL, and GL: final approval of the completed article. All authors contributed to the article and approved the submitted version.

## Conflict of Interest

The authors declare that the research was conducted in the absence of any commercial or financial relationships that could be construed as a potential conflict of interest.
